# Opioid overdose prevention education in Texas during the COVID-19 pandemic

**DOI:** 10.1186/s12954-023-00769-y

**Published:** 2023-03-24

**Authors:** Charles W. Mathias, Diana M. Cavazos, Kelly McGlothen-Bell, Allison D. Crawford, Brieanna Flowers-Joseph, Zhan Wang, Lisa M. Cleveland

**Affiliations:** 1 Department of Psychiatry and Behavioral Sciences, The University of Texas Health Science Center at San Antonio, 7703 Floyd Curl Drive, MC 7793, San Antonio, TX 78229 USA; 2 School of Nursing, The University of Texas Health Science Center at San Antonio, San Antonio, USA; 3 Graduate School of Biomedical Sciences, The University of Texas Health Science Center at San Antonio, San Antonio, USA; 4 Population Health Sciences, The University of Texas Health Science Center at San Antonio, San Antonio, USA

**Keywords:** Training, Naloxone, Overdose reversal, Opioids, COVID-19

## Abstract

**Background:**

Distribution of naloxone and training on its proper use are evidence-based strategies for preventing opioid overdose deaths. In-person naloxone training was conducted in major metropolitan areas and urban centers across Texas as part of a state-wide targeted opioid response program. The training program transitioned to a live, virtual format during the COVID-19 public health emergency declaration. This manuscript describes the impact of this transition through analyses of the characteristics of communities reached using the new virtual training format.

**Case presentation:**

Training participant addresses were compared to county rates of opioid overdose deaths and broadband internet access, and census block comparison to health services shortages, rural designation, and race/ethnicity community characteristics.

**Conclusions:**

The virtual training format reached more learners than the in-person events. Training reached nearly half of the counties in Texas, including all with recent opioid overdose deaths. Most participants lived in communities with a shortage of health service providers, and training reached rural areas, those with limited broadband internet availability, and majority Hispanic communities. In the context of restrictions on in-person gathering, the training program successfully shifted to a live, online format. This transition increased participation above rates observed pre-pandemic and reached communities with the need for equipping those most likely to witness an opioid overdose with the proper use of naloxone.

## Background

The US invests significant resources in opioid prevention, treatment, and recovery services to counter the opioid epidemic. The State Targeted Response to the Opioid Crisis [1] and Opioid Response [2] Grants fund the implementation of evidence-based services addressing the harms of opioid use. A leading strategy for overdose prevention is targeted naloxone distribution and training [3–5]. Naloxone is an antagonist that rapidly reverses opioid-induced respiratory depression and hypotension that can cause death. Access to naloxone and training on its administration is a public health intervention demonstrated to reduce the incidence of opioid overdose fatalities [6]. Because naloxone is rarely self-administered, overdose training most commonly supports reversal by a bystander encountering an overdose [7]. As states legalized naloxone distribution to laypersons, overdose fatalities declined by 14%, and these effects were even greater for Black and Hispanic communities [8].

Naloxone distribution strategies focus on equipping those most likely to witness an opioid overdose with the proper use of naloxone. Target audiences include first responders, family, peers, and people who use opioids [9, 10]. To prepare laypersons, distribution is accompanied by training on recognizing an opioid overdose, administering naloxone, and providing basic first aid [9, 11]. Layperson training has demonstrated effectiveness for opioid overdose reversal; in one report, 11% of laypersons administered naloxone within 3 years of training and were successful in overdose reversal in 98% of cases [7].

The COVID-19 pandemic hampered overdose prevention efforts like naloxone distribution and training. Rising opioid deaths in the decade before the pandemic escalated during the public health emergency declaration; US overdose deaths rose nearly 60% in May 2020 [12, 13], and emergency medical services (EMS) calls and EMS naloxone administration significantly increased after COVID-19 [14]. Increases in opioid overdose during the pandemic stemmed from a range of social and economic factors. Restrictions on public gatherings and the ensuing social isolation acted as antecedents to the escalation in substance use and overdoses [15, 16]. Supply chain disruption changed the illicit drug market resulting in exposure to high-potency fentanyl [15]. Workforce and resource restrictions reduced access to harm reduction and medication-assisted services [17]. These service interruptions were most pronounced in underserved communities, especially rural areas [15].

Before the COVID-19 pandemic, naloxone training was typically delivered through live, in-person instruction [18]. Overdose prevention training programs that continued service during the pandemic modified the delivery format to accommodate social distancing regulations. For instance, delivery shifted from indoors and group to outdoors and individual online training, which reached 1,539 trainees during the first 10 months of the pandemic in West Virginia, although no comparison to pre-pandemic training rates was reported [19]. An Ohio program increased training and distribution after the pandemic onset by shifting from in-person training to “drive-through” events (up to 172% increase), individual telephone consultations (265% increase), and online training that created a low level of distribution reaching new communities [18]. Online overdose prevention training has been shown to be as effective as in-person, at least for medical personnel [20].

There remains a limited understanding of the impact of overdose training modalities [7]. More information is needed to understand the impact of the sudden changes in evidence-based practices of naloxone training in response to the COVID-19 pandemic. This manuscript describes opioid overdose prevention training conducted as part of the state of Texas Targeted Opioid Response program and in the context of the COVID-19 public health emergency. The reach of training is described relative to location-defined factors associated with opioid mortality and/or barriers to opioid overdose prevention, including comparisons with measures of overdose rates, rurality, health service shortage designation, broadband internet access, and demographics.

## Case presentation

### Training

Overdose Education and Prevention Trainings were conducted in a live, virtual format via Zoom (Zoom^©^ 2022 Video Communications, Inc; San Jose, CA). The training objectives were: (1) Explain how different opioids affect the body and similarities/differences by formulation; (2) Define three major risk factors for opioid overdose; (3) Recognize the signs of an opioid overdose; (4) Respond to an opioid overdose using naloxone/Narcan; (5) Describe the Texas laws about naloxone/Narcan access and administration; (6) Describe the relevance of polysubstance use; and (7) Recognize and respond to a stimulant overdose. Upon completion of training, participants were instructed on how to obtain free Narcan from Texas providers and residents through our distribution website [21].

The training was delivered in English and Spanish language by one of the authors (DMC), an expert in harm reduction, multicultural and migrant health. Training in the 2-h live, online format was delivered between November 30, 2020, and March 3, 2022. This format was adopted in response to the restrictions resulting from the COVID-19 public health emergency. Before this shift to virtual training, in-person trainings were conducted: counts from the 16 months prior to the online programming are reported to compare training volume. Because the onsite training used a different registration system, we cannot compare the two training formats on other factors of interest (e.g., county, health service shortage area, etc.).

### Enrollment

Recruitment for the training was conducted through online and direct email advertisements. Training advertisements described the goal of “Decreasing the adverse impact of opioids on Texas residents, with an immediate emphasis on reducing overdose mortality through best practices and providing greater access to opioid overdose medication such as Naloxone/Narcan.” Session content included: “Updates on current trends driving increases in overdose frequency and mortality. A framework for understanding the complex individual, social, and situational factors that shape overdose risk. Actions we can take to strengthen our efforts at preventing overdose frequency. Instruction on recognizing and responding to an overdose.” Online advertisements were posted at the program website [21], the Texas Health and Human Services Commission (TX HHSC) funded the University of Texas (UT) Health San Antonio School of Nursing (SON) naloxone distribution and overdose prevention education program. These advertisements were also linked to the Texas Neonatal Abstinence Syndrome Symposium [22] through the UT Health San Antonio Department of Lifelong Learning [23]. The NAS Symposium, also funded by the TX HHSC, is an annual conference that provides education about maternal substance use and Neonatal Abstinence Syndrome/Newborn Opioid Withdrawal Syndrome (NAS/NOWS). This English and Spanish language email marketing campaign was distributed to a list of approximately 10,000 email addresses (Constant Contact Inc., Waltham, MA) from previous registrations to the Department of Lifelong Learning courses, the continuing education program of the SON.

### Registration

Participant registration was conducted using the REDCap electronic data capture system hosted under an end-user agreement with UT Health San Antonio [24, 25]. REDCap (Research Electronic Data Capture) is a secure, web-based software platform designed to support data capture for research studies, providing (1) an intuitive interface for validated data capture; (2) audit trails for tracking data manipulation and export procedures; (3) automated export procedures for seamless data downloads to common statistical packages; and (4) procedures for data integration and interoperability with external sources. During the enrollment process, data were collected about participant demographics (gender, race/ethnicity, age) and their address for analyses of training dissemination.

### Data analyses

Participant addresses were analyzed at county and census tract levels for comparison with local health outcome measures. At the county level, comparisons were made with opioid overdoses occurring in the 3 years prior to training [26] and to county rates of internet broadband access [27]. Spearman’s ρ was used to test the relationship between the density of training by county opioid overdoses and broadband access level.

Participant addresses were identified and converted to geographic coordinates (latitude and longitude) using the Geocod.io RESTful API (Dotsquare, LLC., Virginia Beach, VA). The Geocod.io returns a score for locations ranging from 0.00 to 1.00 based on confidence in location accuracy; scores of > 0.80 are interpreted as acceptable accuracy [28]. Of the 1901 addresses, 1823 were accurate; these were cross-referencing for health service shortage designation, 1230 cases were analyzed for rural designation (593 failed to match rural location), and 1639 cases were included for race/ethnicity (184 failed to match). Specifically, linkages were made to Health Professional Shortage Areas (HPSA) for Mental Health and Medically Underserved Areas/Populations [29]. HPSAs for Mental Health are locations designated by the Health Resources & Services Administration as having a shortage of mental health professionals, while MUA/P are designated as having a shortage of primary care professionals [30]. The rural designation was computed from the Rural–Urban Commuting Area (RUCA) codes; primary RUCA scores > 4 were interpreted as rural [31]. The proportion of race/ethnicity was extracted against demographic data from the American Community Survey [32].

Statistical tests were conducted using SPSS version 28 (IBM Corp., Armonk, NY). These analyses were conducted for program evaluation. The local institutional review board designated this use of data as not regulated research (US HHS 45 CFR 46 and US FDA 21 CFR 56.). Because the analyses focused on address data, to protect the confidentiality of trainees, this dataset has not been uploaded to a repository.

### Outcomes

#### Training attendance

During the first 16 months of the live, virtual training, 82 sessions were delivered across 63 days (on 19 occasions, offered morning and afternoon sessions on the same day). Of the 2861 program registrants, 1982 (69% of registrants) attended the virtual training, 818 (29%) failed to attend, and 61 (2%) canceled registration prior to the training session. The average class size was 24 attendees (range = 4–128 participants). The demographic characteristics of participants appear in Table 1; most learners were women, of white race and Hispanic ethnicity, while the most common age group were 25–34 years. While our marketing efforts were aimed at reaching a broad audience of bystanders, the slightly higher participation rate among women (57.7%) reflects the composition of the mailing lists from the continuing education program of the SON.

**Table 1:** Demographic characterizes of trainees

	#	%
*Gender*		
Women	1142	57.8
Men	307	15.5
Other	8	0.4
Unknown	521	26.3
*Race/ethnicity*		
African American	206	10.4
Am. Indian/Alaska Native	26	1.3
Asian	32	1.6
Hispanic/Latino	601	30.4
White	602	30.4
US pacific Islander	2	0.1
Other race/ethnicity	31	1.6
Unknown race/ethnicity	478	24.2
*Age (years)*		
< 25	144	7.3
25–34	361	18.2
35–44	339	17.1
45–54	312	15.7
55–64	241	12.2
> 64	59	3.0
Unknown	526	26.5

To contextualize virtual training attendance, 1242 people received training across 39 training sessions during the prior 16 months. In other words, there was a 60% increase in participants reached and a 110% increase in training in the virtual format under COVID-19 restrictions. Because the in-person events used a different registration system, further comparison of participant characteristics and location addresses were not available for comparison to virtual training events.

#### Distribution of training

**Fig. 1:**
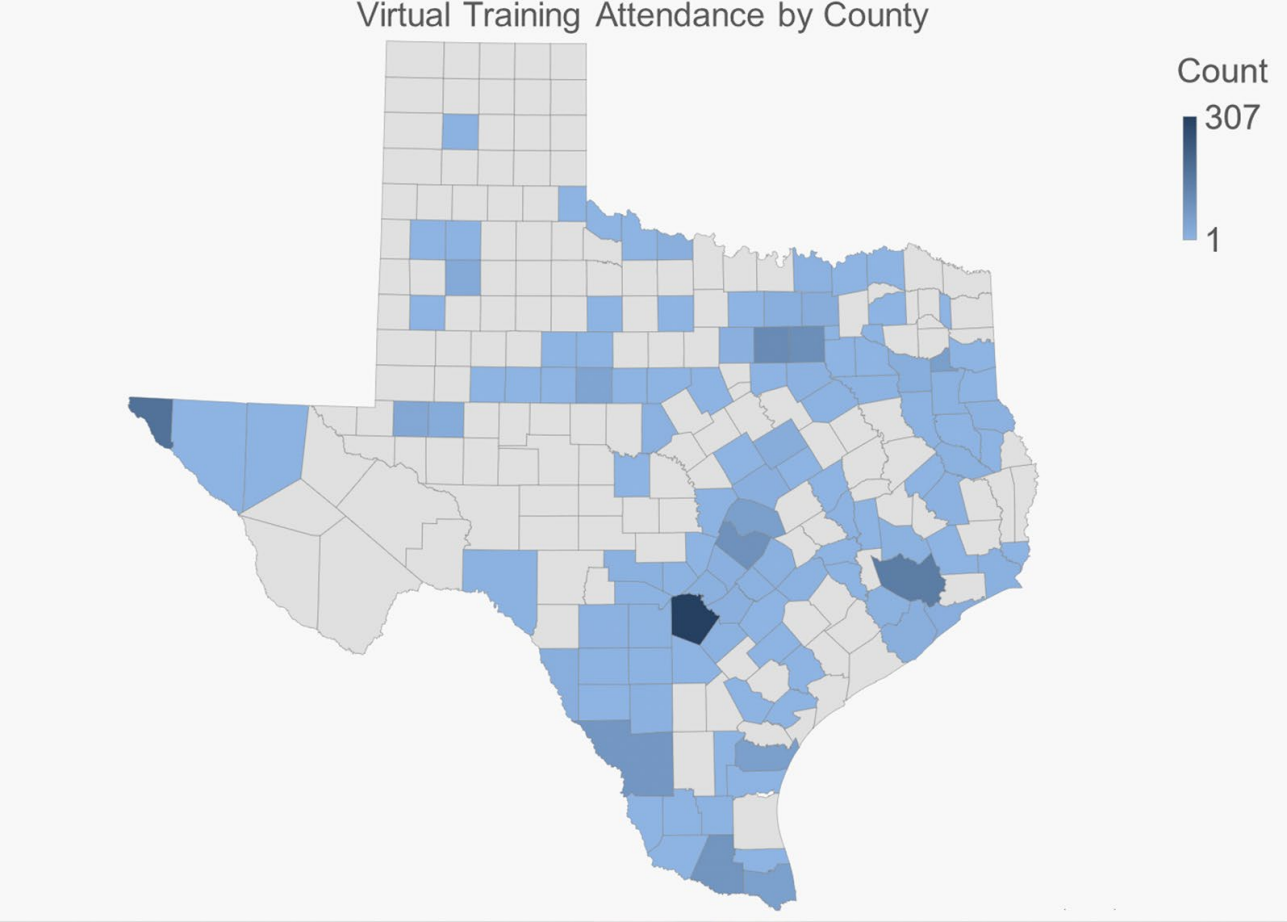
Frequency of virtual training attendance by county

Participant addresses were reported by 1901 (96%) participants. The virtual training format offered the ability to reach a broad audience representing 115 (45.3%) of Texas’ 254 counties. Figure 1 shows the distribution of counties with attendees (darker blue reflects more attendees from that county). Another 32 people participated from locations outside of Texas; 6 from Puerto Rico; 5 from NM; 2 participants each from AR, CA, MI, NY; 1 each from GA, IL, KY, LA, MA, MN, MO, MT, NE, OH, TN, VA, and Ontario, Canada.

##### Overdoses

Training reached participants in 100% of Texas counties reporting opioid overdoses in the two preceding years. Between 2018 and 2020, 4591 opioid overdoses were reported in the state of Texas across 178 counties. A total of 1968 (99.2%) of trainings reached counties with overdose deaths. There was a significant association between the frequency of training attendance and the number of opioid overdoses (Spearman’s *ρ* = .473, *p* < .001). Those counties with more overdoses had more participants attending the training.

##### Broadband access

Relatively more training was delivered to counties with higher broadband access. However, the program reached counties with lower broadband access: 18.5% of counties below the state median, and 5.4% were those in the lowest state quartile for broadband access received training. In Texas in 2021, the median county broadband access rate was 74% (Min–Max by County = 46–93%). There was a significant correlation between the number of trainings delivered and the level of broadband access per county (Spearman’s *ρ* = .363, *p* < .001).

##### Rural designation

8.4% of participants were classified as rural. The median RUCA score was 1 (metropolitan).

##### Health service shortage designation

The vast majority (92.3%) of participants were from Health Professional Shortage Areas (HPSA) for Mental Health, and 45.5% resided in Medically Underserved Areas for Primary Care Services.

##### Race/ethnicity designation

Training attendees were from diverse communities across Texas; 44.5% of participants lived in a Hispanic majority community (i.e., > 50% of the county population identifies as white-Hispanic). The average race/ethnicity for participant census tracks was: 42.8% white-Hispanic; 33.2% white-NonHispanic; 9.9% Black, 6.8% Other, 3.7% Asian, 1.7% two or more races and NonHispanic, 1.2% two or more races and Hispanic, 0.6% American Indian, and 0.1% Pacific Islander.

## Discussion

This manuscript describes the opioid overdose prevention training in Texas during the COVID-19 public health emergency. As training transitioned from in-person to live, online events, there was an increase in the number of learners. Previous research suggests that analyses that are more geographically specific than aggregate county level would better inform targeted opioid overdose response because it can account for factors contributing to health disparities in response to opioid overdose [33]. The current analyses consider sub-county and census block-level analyses demonstrating the reach of training to areas with opioid overdose deaths, as well as by rural, health service shortage, low broadband access, and racial/ethnic characteristics.

Texas mirrors the US trends in opioid deaths and poses specific challenges in overdose prevention training. In 2019, 1372 opioid overdose deaths were reported in Texas, up 36% from a decade prior [26]. These deaths were highest in the state’s population centers: 307 deaths in Harris County (Houston), 163 Dallas County (Dallas), 130 Bexar County (San Antonio), 85 Travis County (Austin), and 75 Tarrant County (Fort Worth) [26]. The training program successfully reached all counties reporting opioid overdoses, and the quantity of these trainings was higher in those counties with more overdose deaths. Training bystanders to recognize and respond to opioid overdose has demonstrated efficacy in reducing fatalities; more trainings are related to greater reductions in deaths within that geographic area [6]. Overdose prevention training exerts a community impact, because training outcomes affect not only the trainee but any overdose they may encounter [6]. This significant relationship is one approach to “saturation”: focusing prevention training on those communities with high overdose death rates. This is a strategy currently prioritized in the Substance Abuse and Mental Health Services Administration's State Opioid Response programming [34].

Beyond the urban centers, the vast geography of Texas poses a unique challenge in implementing opioid overdose prevention training. Texas is the second most populous state (population of approximately 29,527,941; as of 2021; [35]) and the second largest state, with a land mass of 261,231.7 square miles [36]. Scaling opioid prevention training requires addressing rurality, health service shortages, low broadband access, and the unique racial/ethnic characteristics of the 254 counties within the state's boundaries. Nearly half of the state's counties were reached during the first 16 months of online training. Slightly more than 8% of training participants lived in rural communities; to put that in perspective, 10% of Texas residents are rural.

One of the challenges of living outside urban centers is the reduced access to healthcare. Compared to their urban counterparts, people living in rural communities are more likely to experience chronic diseases, diminished access to healthcare, and worse health outcomes [37]. The Health Resources & Services Administration designates areas with an inadequate supply of primary care, dental, and mental health providers within a particular geographic area [30]. In Texas, 70% of counties are health service shortage areas, and 87% are medically underserved for primary care [38]. Substance use services are provided in primary care and mental health settings [39, 40]. People living in counties with fewer primary care and mental health providers have a higher risk of opioid overdose [41], face significant delays in emergency medical services in the event of an overdose [33], and are less likely to be administered naloxone during an overdose [42]. Opioid overdose training presents an opportunity to fill the gap in access to trained opioid healthcare providers. With insufficient access to medical services, the local population is the first responder. In the current evaluation, most participants had an inadequate number of mental health professionals, and nearly half lived with a shortage of primary care providers. One criticism of overdose prevention training evaluation has been the failure to consider the availability of local healthcare capacity for responding to an opioid overdose [6]; the current analyses address this gap by comparing learner location with health services shortage areas. Future studies should evaluate the contextual indicators of implementation success in HPSAs. Addressing this knowledge gap would provide a foundation for replicating targeted training specific to the needs of under-resourced areas.

Telehealth can be a solution for care delivery in rural and health service shortage areas [43]. Telehealth and remote health training require sufficient internet speed (typically 25Mbs/sec) and rural/remote areas are less likely to have the physical infrastructure necessary to support broadband internet [44]. Communities without broadband internet experience barriers in access to health information that telehealth is intended to overcome. Deficiencies in broadband internet access hinder the ability to retrieve credible information related to health and healthcare, which can exacerbate disparities for those living in these communities [45]. Variations in broadband internet access and its influence on the social determinants of health became more evident during the pandemic, as telehealth barriers were concentrated among vulnerable groups [45, 46]. Innovative strategies for increasing access to overdose prevention training are needed to reach the everchanging healthcare landscape for those with opioid use disorder. Rural disparities in broadband internet present another barrier to Texans’ overdose prevention education and training. Approximately 18% of Texas households lack broadband access, and in some rural areas, less than half the population has high-speed internet [27]. In the current analyses, we found that online trainings reached counties with low broadband internet availability; nearly 1 in 5 trainees were in communities below the state's rate for community broadband availability.

In addition to its vast geographic space, Texas reflects substantial ethnic, racial, and cultural diversity. This training program reached counties characterized by high rates of Hispanic residents; nearly half of the counties reached were Hispanic majority populations. Provisioning overdose prevention services to the Hispanic community is especially relevant in Texas, where 40% of people identify as Hispanic, and this group accounts for 65% of the state population growth [47]. Between 1999 and 2020, opioid overdose deaths increased by 328% among Hispanic Texans (from 1.4 to 4.6/100,000; [48]). About 12% of US opioid overdoses occur among Hispanics, but in Texas, they account for 26% of fatalities [48]. In the context of this demography, it is imperative to understand the cultural aspects of the Hispanic community to make harm reduction strategies equally accessible and effectively implemented. One cultural consideration is the value the Hispanic community places on *Familismo*; the mutual obligation, reciprocity, and solidarity to family [49]. *Familismo* can act as a risk- (e.g., the inter-generational transition of substance use) and a protective- (e.g., reduced bi-cultural stress) factor for substance use and overdose [50]. Without tailored communication and cultural competence, addressing opioid use among the Hispanic community will be ineffective. It is essential to consider the cultural tailoring of overdose prevention training for Hispanic communities.

This program's outcomes should be interpreted in the context of the COVID-19 pandemic. Other programs are starting to report their experiences under the many and varied constraints posed by the pandemic. For instance, a rural Appalachian community-based coalition reported on transitioning from in-person overdose trainings to one-on-one drive-thru instruction and video recordings. These adaptations increased the number of persons trained, reduced stigma, and were less costly than traditional in-person methods [18]. Another study of women in South Central Texas used online, virtual check-ins, counseling sessions, and symptom management strategies during the pandemic [51]. Mothers with substance use disorder reported that virtual options for their medication assistance therapy made it easier to balance their responsibilities as caregivers and obligations with their employers and helped them successfully fulfill the conditions of their probation [51]. The barriers associated with transportation, childcare, and time off work were alleviated when virtual options were offered [51].

### Limitations

Interpretation of this program description should consider the context and limitations it was designed under. First, this program was not designed as a research study and lacked the controls inherent in designs such as the randomized control trial. Second, the program did not collect data on overdose reversals or administration of naloxone by program participants, which precluded interpretation of the effectiveness of the training program. Third, the registration process did not collect information about training participants' own substance use. Fourth, this manuscript describes program experiences in Texas, which may not generalize to other states or contexts. Fifth, the pre-pandemic registration process did not include data collection necessary for spatial analyses and precluded comparison with training reach during the pandemic.

## Conclusions

This report describes Texas naloxone training in the context of the COVID-19 pandemic. The program successfully shifted to a live, online format, which increased training participation and reached urban metro population centers where opioid overdose death rates are highest, rural areas with health service shortages, low rates of high-speed internet access, and substantial racial/ethnic diversity. Virtual outreach will likely remain an essential modality for delivering prevention training during and after the COVID-19 pandemic [52].

## Data Availability

The datasets generated and/or analyzed for this project study are not publicly available. The primary data are personal addresses; to protect the privacy of program participants, their information is not shared.

## Abbreviations

HHS: US Department of Health and Human Services
HPSA: Health Professional Shortage Areas
KFF: Kaiser Family Foundation
MUA/P: Mental Health and Medically Underserved Areas/Populations
NAS: Neonatal Abstinence Syndrome
NOWS: Newborn Opioid Withdrawal Syndrome
REDCap: Research Electronic Data Capture
RUCA: Rural–Urban Commuting Area
TX HHSC: Texas Health and Human Services Commission
